# Detecting structural variants involving repetitive elements: capturing transposition events of IS elements in the genome of *Escherichia coli*

**DOI:** 10.1186/1471-2105-13-S18-A12

**Published:** 2012-12-14

**Authors:** Heewook Lee, Ellen Popodi, Patricia L Foster, Haixu Tang

**Affiliations:** 1 School of Informatics and Computing, Indiana University, Bloomington, IN, USA; 2 Department of Biology, Indiana University, Bloomington, IN, USA

## Background

Discordant read pairs [1,2] – those deviating either from expected insert size range or correct relative orientation – have served as vital clues to identifying structural variants (SV) in genomes. Collecting discordant read pairs is the first step in SV detection and is often done by sequence alignment. When there are repetitive elements, such as insertion sequence (IS), a class of transposable elements in bacterial genomes, discordant read pairs can have multiple mapping loci – making them more challenging to be placed and interpreted. Instead of resolving such tangled mapping results, many tools simply ignore these mapped read pairs, potentially missing SVs involving repetitive elements.

## Methods

We present an idea of using approximate *de Bruijn* graphs (*A*-Bruijn graphs) [3] to identify discordant read pairs, in order to discover SVs. Repeats are easily recognized in *A*-Bruijn graphs, as all repetitive elements of the same kind are collapsed into a contiguous edge. When read pairs representing repetitive elements are mapped to a reference *A*-Bruijn graph, only those from novel insertions are flagged as discordant and the rest – those from preexisting insertion loci – mapped concordantly.

We applied this approach to whole genome sequencing data [4] (~100x per sample using 90bp x 2 paired end Illumina sequencing) obtained from 38 lines of *Escherichia coli* PFM2, a derivative strain of *E. coli* K-12 MG1655, and 34 lines of a mismatch repair deficient (deletion of *mutL*) derivative that were propagated for ~3,080 and ~375 generations respectively via a mutation accumulation (MA) strategy. All of the inferred IS insertions were directly confirmed by PCR experiments.

## Results

A total of 27 IS transpositions has been detected and includes 5 out of 12 IS families present in *E. coli* K-12. We have also identified an insertion of IS186 that is fixed among all MA lines and not present in the reference *E. coli* genome. 24 out 27 inferred insertions were validated by PCR and 3 of them are currently under analysis. The fixed insertion of IS186 in the samples was also confirmed by PCR.

## Conclusion

Our method can pinpoint SVs by identifying discordant read pairs resulting from novel insertions of repetitive elements, where many other currently available tools fail. This result serves as a first step towards inferring the neutral rate of IS transposition in bacterial genomes.
